# Crystal structure of 6-amino-4-(3-bromo-4-meth­oxy­phen­yl)-3-methyl-2,4-di­hydro­pyrano[2,3-*c*]pyrazole-5-carbo­nitrile dimethyl sulfoxide monosolvate

**DOI:** 10.1107/S2056989015010543

**Published:** 2015-06-06

**Authors:** Sammer Yousuf, Huma Bano, Munira Taj Muhammad, Khalid Mohammed Khan

**Affiliations:** a H.E.J. Research Institute of Chemistry, International Center for Chemical and Biological Sciences, University of Karachi, Karachi 75270, Pakistan

**Keywords:** crystal structure, pyrazole derivative, hydrogen bonding

## Abstract

In the pyrazole mol­ecule of the title solvate, C_15_H_13_BrN_4_O_2_·C_2_H_6_OS, the dihedral angle between the benzene ring and the mean plane of the di­hydro­pyrano[2,3-*c*]pyrazole ring system [r.m.s deviation = 0.031 (2) Å] is 86.71 (14)°. In the crystal, the pyrazole mol­ecules are linked by N—H⋯N hydrogen bonds, forming a layer parallel to (10-1). The pyrazole and dimethyl sulfoxide mol­ecules are connected by an N—H⋯O hydrogen bond.

## Related literature   

For the applications and biological activities of pyrazole derivative, see: Balbia *et al.* (2011 ▸); Insuasty *et al.* (2010 ▸); Szabó *et al.* (2008 ▸); Perchellet *et al.* (2006 ▸); Tanitame *et al.* (2004 ▸, 2005 ▸); Abadi *et al.* (2003 ▸). For crystal structures of related compounds, see: Sharma *et al.* (2014 ▸).
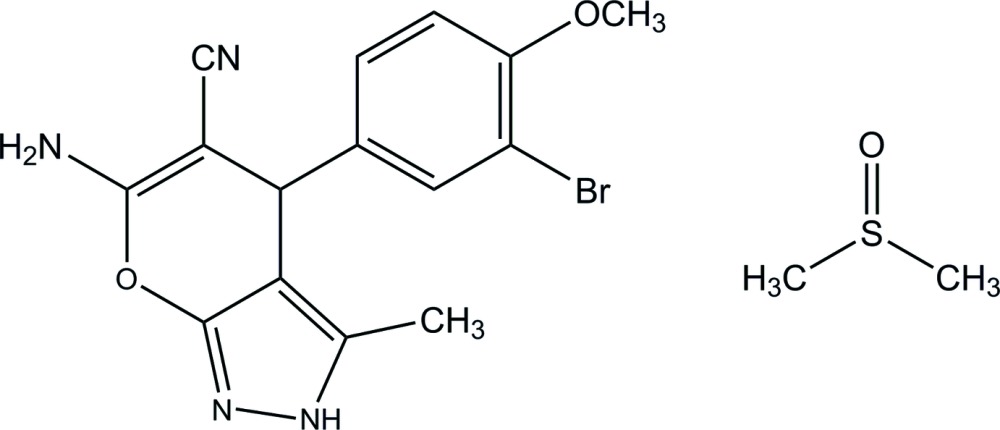


## Experimental   

### Crystal data  

C_15_H_13_BrN_4_O_2_·C_2_H_6_OS
*M* *_r_* = 439.33
Monoclinic, 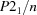

*a* = 13.4982 (6) Å
*b* = 8.3470 (4) Å
*c* = 17.7173 (8) Å
β = 101.510 (1)°
*V* = 1956.05 (16) Å^3^
*Z* = 4
Mo *K*α radiation
μ = 2.23 mm^−1^
*T* = 273 K
0.54 × 0.51 × 0.33 mm

### Data collection  

Bruker SMART APEX CCD area-detector diffractometer
Absorption correction: multi-scan (*SADABS*; Bruker, 2000 ▸) *T*_min_ = 0.345, *T*_max_ = 0.479
9464 measured reflections
3642 independent reflections
2776 reflections with *I* > 2σ(*I*)
*R*_int_ = 0.019

### Refinement  

*R*[*F* ^2^ > 2σ(*F* ^2^)] = 0.052
*wR*(*F* ^2^) = 0.149
*S* = 1.04
3642 reflections
247 parameters
H atoms treated by a mixture of independent and constrained refinement
Δρ_max_ = 1.15 e Å^−3^
Δρ_min_ = −0.59 e Å^−3^

### 

Data collection: *SMART* (Bruker, 2000 ▸); cell refinement: *SAINT* (Bruker, 2000 ▸); data reduction: *SAINT*; program(s) used to solve structure: *SHELXS97* (Sheldrick, 2008 ▸); program(s) used to refine structure: *SHELXL97* (Sheldrick, 2008 ▸); molecular graphics: *SHELXTL* (Sheldrick, 2008 ▸); software used to prepare material for publication: *SHELXTL*, *PARST* (Nardelli, 1995 ▸) and *PLATON* (Spek, 2009 ▸).

## Supplementary Material

Crystal structure: contains datablock(s) global, I. DOI: 10.1107/S2056989015010543/is5403sup1.cif

Structure factors: contains datablock(s) I. DOI: 10.1107/S2056989015010543/is5403Isup2.hkl

Supporting information file. DOI: 10.1107/S2056989015010543/is5403Isup3.cml

**Fig. 1.**
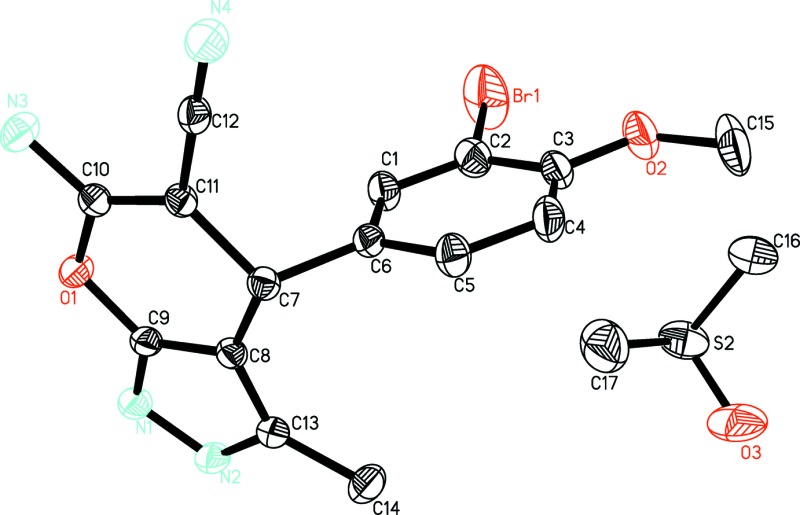
The molecular structure of the title compound with displacement ellipsoids drawn at 30% probability level. H atoms have been omitted.

. DOI: 10.1107/S2056989015010543/is5403fig1.tif
The mol­ecular structure of the title compound with displacement ellipsoids drawn at 30% probability level. H atoms have been omitted.

**Fig. 2.**
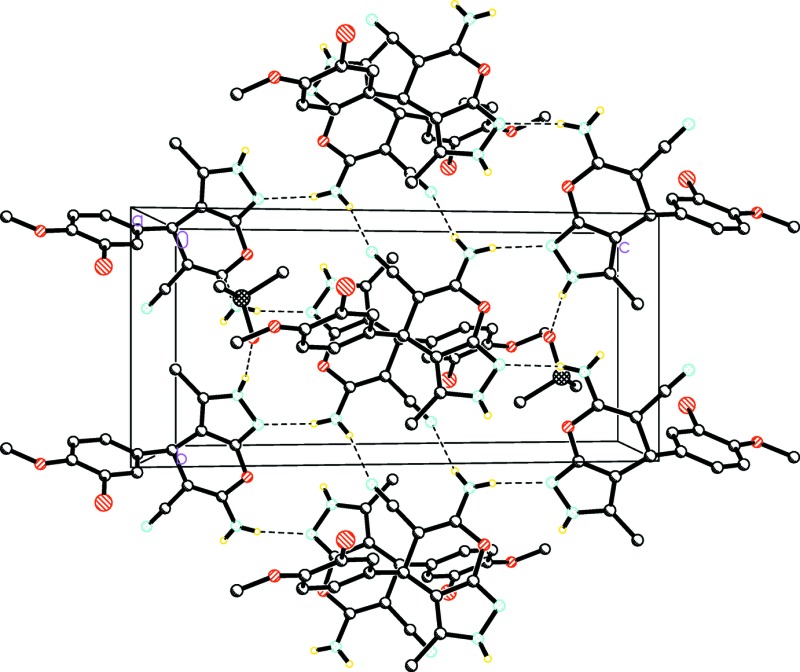
A crystal packing view of the title compound. Only H atoms involved in the hydrogen bonds (dashed lines) are shown.

. DOI: 10.1107/S2056989015010543/is5403fig2.tif
A crystal packing view of the title compound. Only H atoms involved in the hydrogen bonds (dashed lines) are shown.

CCDC reference: 1404448

Additional supporting information:  crystallographic information; 3D view; checkCIF report

## Figures and Tables

**Table 1:** Hydrogen-bond geometry (, )

*D*H*A*	*D*H	H*A*	*D* *A*	*D*H*A*
N2H2*A*O3^i^	0.82(3)	1.96(4)	2.762(5)	166(4)
N3H3*A*N4^ii^	0.84(4)	2.26(4)	3.080(5)	165(3)
N3H3*B*N1^iii^	0.86(3)	2.14(4)	2.983(4)	169(3)

Symmetry codes: (i) 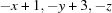; (ii) 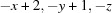; (iii) 
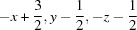
.

## supplementary crystallographic information

### S1. Comment

The pyrazole moiety containing compounds represent an important group of pharmaceutically active molecules with a wide range of biological activities including antifungal (Tanitame *et al.*, 2004), antibacterial (Tanitame *et al.*, 2005), anti-diabetic, (Balbia *et al.*, 2011), anti-inflammatory (Szabo *et al.*, 2008) and antiangiogenesis (Abadi *et al.*, 2003). The pyrazole derivatives are also known to have antiproliferative (Perchellet *et al.*, 2006) and anti-tumor (Insuasty *et al.*, 2010) activities. The title compound was synthesize as a part of our ongoing research to synthesize and evaluate the biological activities of structural analogues of dihydropyrano[2,3-*c*] pyrazole derivatives. In continuation of our efforts to purify enantiomerically pure compounds from racemic mixtures by using simple crystallization techniques the title compound was crystallize as dimethyl sulfoxide (DMSO) solvate from racemic mixture by dissolving in DMSO at room temperature.

The structure of title compound is similar to that of previously published 6-amino-3-methyl-4-(3,4,5-trimethoxy-phenyl)-2,4-dihydropyrano[2,3-*c*]- pyrazole-5-carbonitrile (Sharma *et al.*, 2014) with the difference that trimethoxy sustituted phenyl ring is replaced by methoxy substituted bromo benzene ring (Fig. 1). The dihedral angles between the benzene (C1–C6) / pyran (O1/C7–C9/C10/C11) rings and the benzene (C1–C6) / pyrazole (N1/N2/C8/C9/C13) rings are 87.53 (15) and 86.04 (18)°, respectively. The bond lengths and angles are similar as in structurally related benzohydrazide derivatives (Sharma *et al.*, 2014). The crystal structure stabilize by intermolecular N—H···O and N—H···N interactions to form a layer parallel to (101) (Table 2 and Fig. 2).

### S2. Experimental

The title compound was synthesized as follows. Dichloromethane (10 ml), 1.0 equivalent (1 mmol) of triethylamine and pyrazolone were taken in a round bottom flask and allowed to stirred for 2 minutes at room temperature followed by the addition of 1.0 equivalent of corresponding pre-synthesized benzylidene from malononitrile and allowed to stir for additional 25–30 min. The progress of reaction was monitored by TLC. The desired product was appeared in the form of precipitates. The precipitates were washed with water to remove the unreacted pyrazolone to obtain pure products. Yeild 79%; *m*.*p*. 215 °C. The precipitates were redissolved in DMSO and allow to stand at room temperature for whole night followed by the removal of DMSO under freeze drying condition to obtain single crystals suitable for X-ray diffraction.

### S3. Refinement

H atoms on methyl, phenyl and methine groups were positioned geometrically with C—H = 0.96, 0.93 and 0.98 Å, respectively, and constrained to ride on their parent atoms with *U*_iso_(H) = 1.5*U*_eq_(C) for the methyl H atoms or 1.2*U*_eq_(C) for the other H atoms. H atoms on N were located in a difference Fourier map and refined freely [N—H = 0.81 (3)–0.86 (4) Å].

### Crystal data

**Table:**

C_15_H_13_BrN_4_O_2_·C_2_H_6_OS	*F*(000) = 896
*M**_r_* = 439.33	*D*_x_ = 1.492 Mg m^−^^3^
Monoclinic, *P*2_1_/*n*	Mo *K*α radiation, λ = 0.71073 Å
Hall symbol: -P 2yn	Cell parameters from 3172 reflections
*a* = 13.4982 (6) Å	θ = 2.4–25.5°
*b* = 8.3470 (4) Å	µ = 2.23 mm^−^^1^
*c* = 17.7173 (8) Å	*T* = 273 K
β = 101.510 (1)°	BLOCK, colorless
*V* = 1956.05 (16) Å^3^	0.54 × 0.51 × 0.33 mm
*Z* = 4	

### Data collection

**Table:**

Bruker SMART APEX CCD area-detector diffractometer	3642 independent reflections
Radiation source: fine-focus sealed tube	2776 reflections with *I* > 2σ(*I*)
Graphite monochromator	*R*_int_ = 0.019
ω scan	θ_max_ = 25.5°, θ_min_ = 1.7°
Absorption correction: multi-scan (*SADABS*; Bruker, 2000)	*h* = −16→8
*T*_min_ = 0.345, *T*_max_ = 0.479	*k* = −10→10
9464 measured reflections	*l* = −16→21

### Refinement

**Table:**

Refinement on *F*^2^	Primary atom site location: structure-invariant direct methods
Least-squares matrix: full	Secondary atom site location: difference Fourier map
*R*[*F*^2^ > 2σ(*F*^2^)] = 0.052	Hydrogen site location: inferred from neighbouring sites
*wR*(*F*^2^) = 0.149	H atoms treated by a mixture of independent and constrained refinement
*S* = 1.04	*w* = 1/[σ^2^(*F*_o_^2^) + (0.0714*P*)^2^ + 2.3987*P*] where *P* = (*F*_o_^2^ + 2*F*_c_^2^)/3
3642 reflections	(Δ/σ)_max_ < 0.001
247 parameters	Δρ_max_ = 1.15 e Å^−^^3^
0 restraints	Δρ_min_ = −0.59 e Å^−^^3^

### Special details

**Table:**

Geometry. All e.s.d.'s (except the e.s.d. in the dihedral angle between two l.s. planes) are estimated using the full covariance matrix. The cell e.s.d.'s are taken into account individually in the estimation of e.s.d.'s in distances, angles and torsion angles; correlations between e.s.d.'s in cell parameters are only used when they are defined by crystal symmetry. An approximate (isotropic) treatment of cell e.s.d.'s is used for estimating e.s.d.'s involving l.s. planes.
Refinement. Refinement of *F*^2^ against ALL reflections. The weighted *R*-factor *wR* and goodness of fit *S* are based on *F*^2^, conventional *R*-factors *R* are based on *F*, with *F* set to zero for negative *F*^2^. The threshold expression of *F*^2^ > σ(*F*^2^) is used only for calculating *R*-factors(gt) *etc*. and is not relevant to the choice of reflections for refinement. *R*-factors based on *F*^2^ are statistically about twice as large as those based on *F*, and *R*- factors based on ALL data will be even larger.

### Fractional atomic coordinates and isotropic or equivalent isotropic displacement parameters (Å^2^)

**Table:**

	*x*	*y*	*z*	*U*_iso_*/*U*_eq_	
Br1	0.53684 (5)	0.80041 (9)	0.10616 (4)	0.1100 (3)	
S2	0.41262 (9)	1.33033 (14)	0.16737 (7)	0.0725 (4)	
O1	0.80217 (17)	0.8729 (2)	−0.16690 (11)	0.0408 (5)	
C11	0.8828 (2)	0.8290 (3)	−0.03464 (16)	0.0318 (6)	
C10	0.8569 (2)	0.7805 (4)	−0.10982 (17)	0.0347 (7)	
C7	0.8596 (2)	0.9926 (3)	−0.00378 (16)	0.0328 (6)	
H7A	0.9235	1.0499	0.0131	0.039*	
N1	0.7159 (2)	1.1133 (3)	−0.19466 (15)	0.0415 (6)	
N3	0.8795 (3)	0.6418 (4)	−0.13885 (18)	0.0499 (8)	
C12	0.9385 (2)	0.7201 (4)	0.01801 (17)	0.0378 (7)	
C8	0.7977 (2)	1.0822 (3)	−0.07006 (16)	0.0328 (6)	
C6	0.8067 (2)	0.9806 (4)	0.06440 (16)	0.0348 (7)	
N2	0.7048 (2)	1.2445 (3)	−0.15155 (16)	0.0432 (7)	
C9	0.7727 (2)	1.0195 (3)	−0.14372 (16)	0.0345 (7)	
C2	0.6640 (3)	0.9018 (5)	0.1172 (2)	0.0530 (9)	
C13	0.7513 (3)	1.2299 (4)	−0.07755 (18)	0.0388 (7)	
C4	0.7998 (3)	1.0387 (5)	0.19660 (19)	0.0527 (9)	
H4A	0.8299	1.0837	0.2436	0.063*	
N4	0.9847 (3)	0.6326 (4)	0.06142 (17)	0.0545 (8)	
C5	0.8497 (3)	1.0446 (4)	0.13548 (18)	0.0458 (8)	
H5A	0.9131	1.0925	0.1423	0.055*	
O2	0.6509 (2)	0.9565 (4)	0.24480 (16)	0.0713 (8)	
C1	0.7129 (3)	0.9085 (4)	0.05641 (18)	0.0439 (8)	
H1B	0.6826	0.8641	0.0093	0.053*	
C14	0.7477 (3)	1.3584 (4)	−0.0197 (2)	0.0588 (10)	
H14A	0.7084	1.4468	−0.0441	0.088*	
H14B	0.8151	1.3940	0.0015	0.088*	
H14C	0.7171	1.3170	0.0208	0.088*	
C15	0.6936 (4)	1.0205 (7)	0.3188 (2)	0.0878 (16)	
H15A	0.6470	1.0061	0.3527	0.132*	
H15B	0.7068	1.1327	0.3140	0.132*	
H15C	0.7557	0.9659	0.3394	0.132*	
C3	0.7068 (3)	0.9673 (4)	0.18880 (19)	0.0499 (9)	
O3	0.4267 (3)	1.5021 (4)	0.1919 (3)	0.1011 (12)	
C16	0.4432 (4)	1.2187 (6)	0.2534 (3)	0.0736 (12)	
H16A	0.3899	1.2292	0.2817	0.110*	
H16B	0.5052	1.2583	0.2840	0.110*	
H16C	0.4511	1.1079	0.2413	0.110*	
C17	0.5216 (5)	1.2847 (8)	0.1298 (4)	0.106 (2)	
H17A	0.5175	1.3380	0.0812	0.159*	
H17B	0.5255	1.1711	0.1226	0.159*	
H17C	0.5808	1.3204	0.1652	0.159*	
H3A	0.913 (3)	0.572 (5)	−0.110 (2)	0.055 (11)*	
H3B	0.850 (3)	0.620 (4)	−0.185 (2)	0.041 (9)*	
H2A	0.670 (3)	1.320 (4)	−0.1703 (19)	0.034 (9)*	

### Atomic displacement parameters (Å^2^)

**Table:**

	*U*^11^	*U*^22^	*U*^33^	*U*^12^	*U*^13^	*U*^23^
Br1	0.0905 (4)	0.1520 (6)	0.1031 (5)	−0.0652 (4)	0.0569 (4)	−0.0322 (4)
S2	0.0579 (6)	0.0701 (7)	0.0833 (8)	0.0025 (5)	−0.0009 (5)	0.0238 (6)
O1	0.0583 (14)	0.0336 (11)	0.0269 (10)	0.0099 (10)	0.0000 (9)	0.0002 (9)
C11	0.0335 (15)	0.0346 (15)	0.0262 (14)	0.0017 (12)	0.0030 (11)	0.0019 (12)
C10	0.0387 (16)	0.0351 (16)	0.0299 (15)	0.0015 (13)	0.0060 (12)	0.0021 (12)
C7	0.0349 (15)	0.0341 (16)	0.0285 (14)	−0.0028 (12)	0.0042 (12)	−0.0005 (12)
N1	0.0535 (16)	0.0364 (14)	0.0329 (13)	0.0044 (12)	0.0042 (12)	0.0039 (11)
N3	0.072 (2)	0.0428 (17)	0.0293 (15)	0.0176 (16)	−0.0038 (14)	−0.0042 (13)
C12	0.0441 (17)	0.0405 (17)	0.0288 (15)	0.0015 (15)	0.0075 (13)	−0.0036 (14)
C8	0.0380 (16)	0.0325 (15)	0.0277 (14)	−0.0033 (12)	0.0063 (12)	0.0010 (12)
C6	0.0414 (17)	0.0341 (16)	0.0286 (15)	0.0038 (13)	0.0064 (12)	0.0007 (12)
N2	0.0552 (18)	0.0322 (15)	0.0413 (16)	0.0082 (13)	0.0077 (13)	0.0058 (12)
C9	0.0425 (17)	0.0305 (15)	0.0302 (15)	−0.0014 (13)	0.0065 (12)	0.0025 (12)
C2	0.056 (2)	0.055 (2)	0.053 (2)	−0.0082 (18)	0.0221 (17)	−0.0011 (17)
C13	0.0484 (18)	0.0331 (16)	0.0360 (16)	−0.0018 (14)	0.0114 (14)	0.0015 (13)
C4	0.066 (2)	0.062 (2)	0.0303 (17)	0.0052 (19)	0.0112 (16)	−0.0061 (16)
N4	0.069 (2)	0.0520 (18)	0.0379 (16)	0.0160 (16)	0.0002 (14)	0.0015 (14)
C5	0.0481 (19)	0.055 (2)	0.0331 (17)	−0.0028 (16)	0.0045 (14)	−0.0050 (15)
O2	0.089 (2)	0.084 (2)	0.0512 (16)	0.0065 (17)	0.0397 (15)	0.0057 (14)
C1	0.0467 (19)	0.0522 (19)	0.0343 (16)	−0.0085 (16)	0.0115 (14)	−0.0051 (14)
C14	0.087 (3)	0.0398 (19)	0.049 (2)	0.0094 (19)	0.014 (2)	−0.0070 (16)
C15	0.105 (4)	0.127 (4)	0.040 (2)	0.034 (3)	0.034 (2)	0.005 (2)
C3	0.066 (2)	0.051 (2)	0.0377 (18)	0.0106 (18)	0.0239 (16)	0.0077 (15)
O3	0.104 (3)	0.0544 (19)	0.151 (4)	0.0290 (18)	0.041 (2)	0.030 (2)
C16	0.070 (3)	0.065 (3)	0.084 (3)	0.003 (2)	0.011 (2)	0.020 (2)
C17	0.119 (5)	0.109 (5)	0.101 (4)	0.031 (4)	0.047 (4)	0.014 (4)

### Geometric parameters (Å, º)

**Table:**

Br1—C2	1.889 (4)	N2—H2A	0.81 (3)
S2—O3	1.499 (4)	C2—C1	1.371 (5)
S2—C16	1.764 (5)	C2—C3	1.397 (5)
S2—C17	1.773 (6)	C13—C14	1.492 (5)
O1—C10	1.365 (4)	C4—C3	1.372 (5)
O1—C9	1.374 (4)	C4—C5	1.385 (5)
C11—C10	1.369 (4)	C4—H4A	0.9300
C11—C12	1.408 (4)	C5—H5A	0.9300
C11—C7	1.527 (4)	O2—C3	1.365 (4)
C10—N3	1.327 (4)	O2—C15	1.426 (6)
C7—C8	1.498 (4)	C1—H1B	0.9300
C7—C6	1.524 (4)	C14—H14A	0.9600
C7—H7A	0.9800	C14—H14B	0.9600
N1—C9	1.319 (4)	C14—H14C	0.9600
N1—N2	1.360 (4)	C15—H15A	0.9600
N3—H3A	0.84 (4)	C15—H15B	0.9600
N3—H3B	0.86 (4)	C15—H15C	0.9600
C12—N4	1.150 (4)	C16—H16A	0.9600
C8—C13	1.377 (4)	C16—H16B	0.9600
C8—C9	1.384 (4)	C16—H16C	0.9600
C6—C1	1.384 (4)	C17—H17A	0.9600
C6—C5	1.385 (4)	C17—H17B	0.9600
N2—C13	1.341 (4)	C17—H17C	0.9600
			
O3—S2—C16	105.1 (2)	C8—C13—C14	130.9 (3)
O3—S2—C17	104.3 (3)	C3—C4—C5	121.0 (3)
C16—S2—C17	98.2 (3)	C3—C4—H4A	119.5
C10—O1—C9	115.3 (2)	C5—C4—H4A	119.5
C10—C11—C12	116.9 (3)	C6—C5—C4	121.1 (3)
C10—C11—C7	125.6 (3)	C6—C5—H5A	119.5
C12—C11—C7	117.4 (2)	C4—C5—H5A	119.5
N3—C10—O1	109.7 (3)	C3—O2—C15	117.6 (4)
N3—C10—C11	126.9 (3)	C2—C1—C6	120.7 (3)
O1—C10—C11	123.3 (3)	C2—C1—H1B	119.6
C8—C7—C6	112.2 (2)	C6—C1—H1B	119.6
C8—C7—C11	106.7 (2)	C13—C14—H14A	109.5
C6—C7—C11	112.7 (2)	C13—C14—H14B	109.5
C8—C7—H7A	108.3	H14A—C14—H14B	109.5
C6—C7—H7A	108.3	C13—C14—H14C	109.5
C11—C7—H7A	108.3	H14A—C14—H14C	109.5
C9—N1—N2	102.0 (2)	H14B—C14—H14C	109.5
C10—N3—H3A	120 (3)	O2—C15—H15A	109.5
C10—N3—H3B	117 (2)	O2—C15—H15B	109.5
H3A—N3—H3B	122 (4)	H15A—C15—H15B	109.5
N4—C12—C11	179.2 (4)	O2—C15—H15C	109.5
C13—C8—C9	103.1 (3)	H15A—C15—H15C	109.5
C13—C8—C7	133.9 (3)	H15B—C15—H15C	109.5
C9—C8—C7	122.9 (3)	O2—C3—C4	125.7 (3)
C1—C6—C5	118.0 (3)	O2—C3—C2	116.5 (4)
C1—C6—C7	120.8 (3)	C4—C3—C2	117.8 (3)
C5—C6—C7	121.2 (3)	S2—C16—H16A	109.5
C13—N2—N1	113.1 (3)	S2—C16—H16B	109.5
C13—N2—H2A	126 (2)	H16A—C16—H16B	109.5
N1—N2—H2A	121 (2)	S2—C16—H16C	109.5
N1—C9—O1	119.2 (3)	H16A—C16—H16C	109.5
N1—C9—C8	114.8 (3)	H16B—C16—H16C	109.5
O1—C9—C8	126.0 (3)	S2—C17—H17A	109.5
C1—C2—C3	121.4 (3)	S2—C17—H17B	109.5
C1—C2—Br1	120.4 (3)	H17A—C17—H17B	109.5
C3—C2—Br1	118.2 (3)	S2—C17—H17C	109.5
N2—C13—C8	106.9 (3)	H17A—C17—H17C	109.5
N2—C13—C14	122.2 (3)	H17B—C17—H17C	109.5
			
C9—O1—C10—N3	179.1 (3)	C7—C8—C9—N1	178.5 (3)
C9—O1—C10—C11	−0.9 (4)	C13—C8—C9—O1	179.3 (3)
C12—C11—C10—N3	−0.7 (5)	C7—C8—C9—O1	−1.9 (5)
C7—C11—C10—N3	177.0 (3)	N1—N2—C13—C8	0.9 (4)
C12—C11—C10—O1	179.4 (3)	N1—N2—C13—C14	−179.0 (3)
C7—C11—C10—O1	−2.9 (5)	C9—C8—C13—N2	−0.4 (3)
C10—C11—C7—C8	4.0 (4)	C7—C8—C13—N2	−179.0 (3)
C12—C11—C7—C8	−178.4 (3)	C9—C8—C13—C14	179.6 (4)
C10—C11—C7—C6	127.6 (3)	C7—C8—C13—C14	0.9 (6)
C12—C11—C7—C6	−54.7 (4)	C1—C6—C5—C4	−0.8 (5)
C6—C7—C8—C13	52.8 (4)	C7—C6—C5—C4	177.6 (3)
C11—C7—C8—C13	176.8 (3)	C3—C4—C5—C6	0.8 (6)
C6—C7—C8—C9	−125.6 (3)	C3—C2—C1—C6	0.1 (6)
C11—C7—C8—C9	−1.6 (4)	Br1—C2—C1—C6	−179.1 (3)
C8—C7—C6—C1	58.8 (4)	C5—C6—C1—C2	0.3 (5)
C11—C7—C6—C1	−61.8 (4)	C7—C6—C1—C2	−178.1 (3)
C8—C7—C6—C5	−119.6 (3)	C15—O2—C3—C4	−2.0 (6)
C11—C7—C6—C5	119.8 (3)	C15—O2—C3—C2	179.1 (4)
C9—N1—N2—C13	−1.0 (4)	C5—C4—C3—O2	−179.2 (3)
N2—N1—C9—O1	−178.8 (3)	C5—C4—C3—C2	−0.4 (6)
N2—N1—C9—C8	0.8 (4)	C1—C2—C3—O2	178.8 (3)
C10—O1—C9—N1	−177.1 (3)	Br1—C2—C3—O2	−2.0 (5)
C10—O1—C9—C8	3.4 (4)	C1—C2—C3—C4	−0.1 (6)
C13—C8—C9—N1	−0.3 (4)	Br1—C2—C3—C4	179.1 (3)

### Hydrogen-bond geometry (Å, º)

**Table:**

*D*—H···*A*	*D*—H	H···*A*	*D*···*A*	*D*—H···*A*
N2—H2*A*···O3^i^	0.82 (3)	1.96 (4)	2.762 (5)	166 (4)
N3—H3*A*···N4^ii^	0.84 (4)	2.26 (4)	3.080 (5)	165 (3)
N3—H3*B*···N1^iii^	0.86 (3)	2.14 (4)	2.983 (4)	169 (3)

Symmetry codes: (i) −*x*+1, −*y*+3, −*z*; (ii) −*x*+2, −*y*+1, −*z*; (iii) −*x*+3/2, *y*−1/2, −*z*−1/2.
