# Supplementary figures and images for: Crystal structure of 6-amino-4-(3-bromo-4-meth­oxy­phen­yl)-3-methyl-2,4-di­hydro­pyrano[2,3-c]pyrazole-5-carbo­nitrile dimethyl sulfoxide monosolvate
Source: Acta Crystallogr E Crystallogr Commun. 2015 Jun 6;71(Pt 7):o453–4. doi: 10.1107/S2056989015010543 (PMC4518963; doi:10.1107/S2056989015010543)

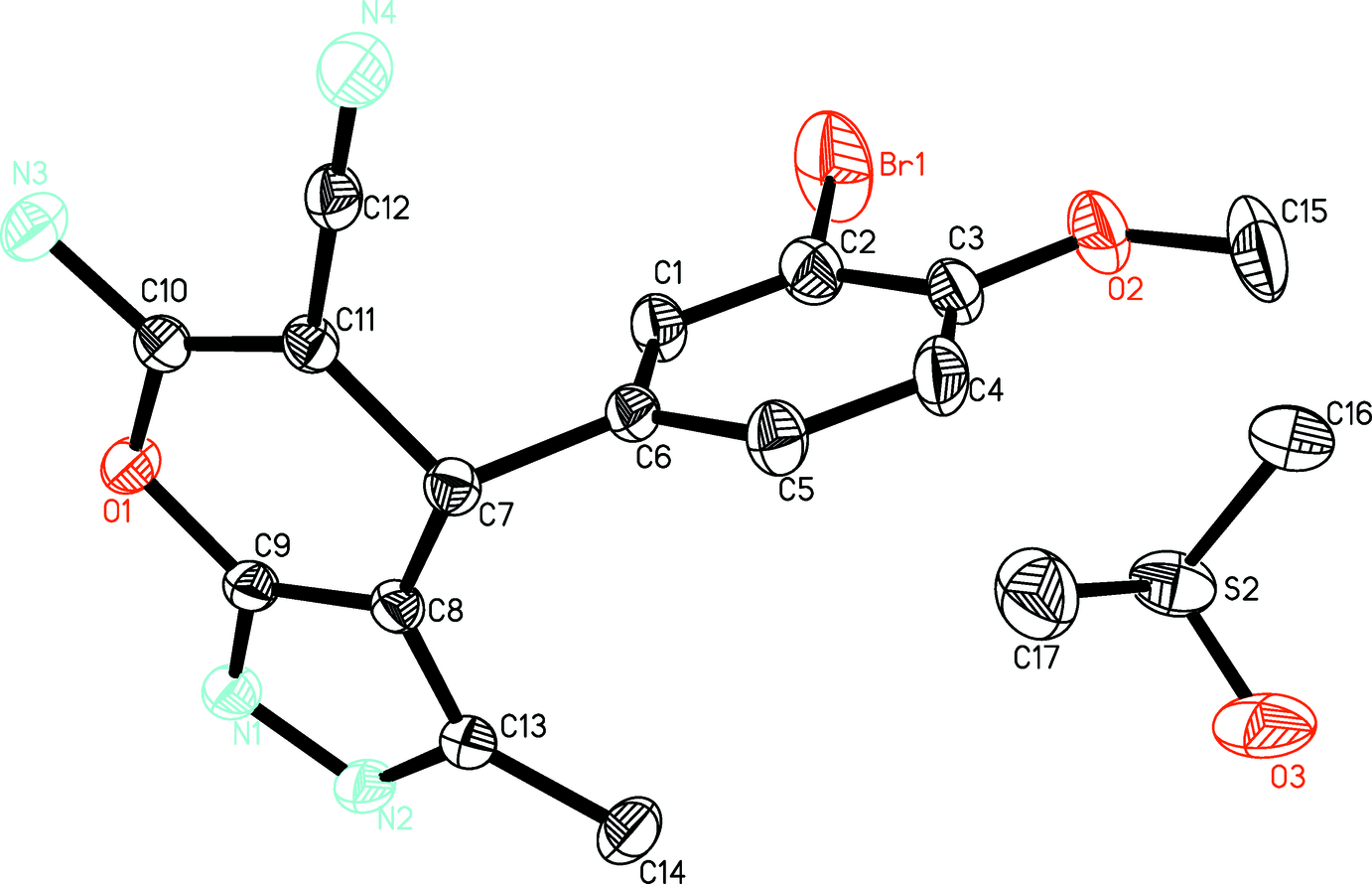

Supplement: Supplementary file 4 [file e-71-0o453-fig1.tif]

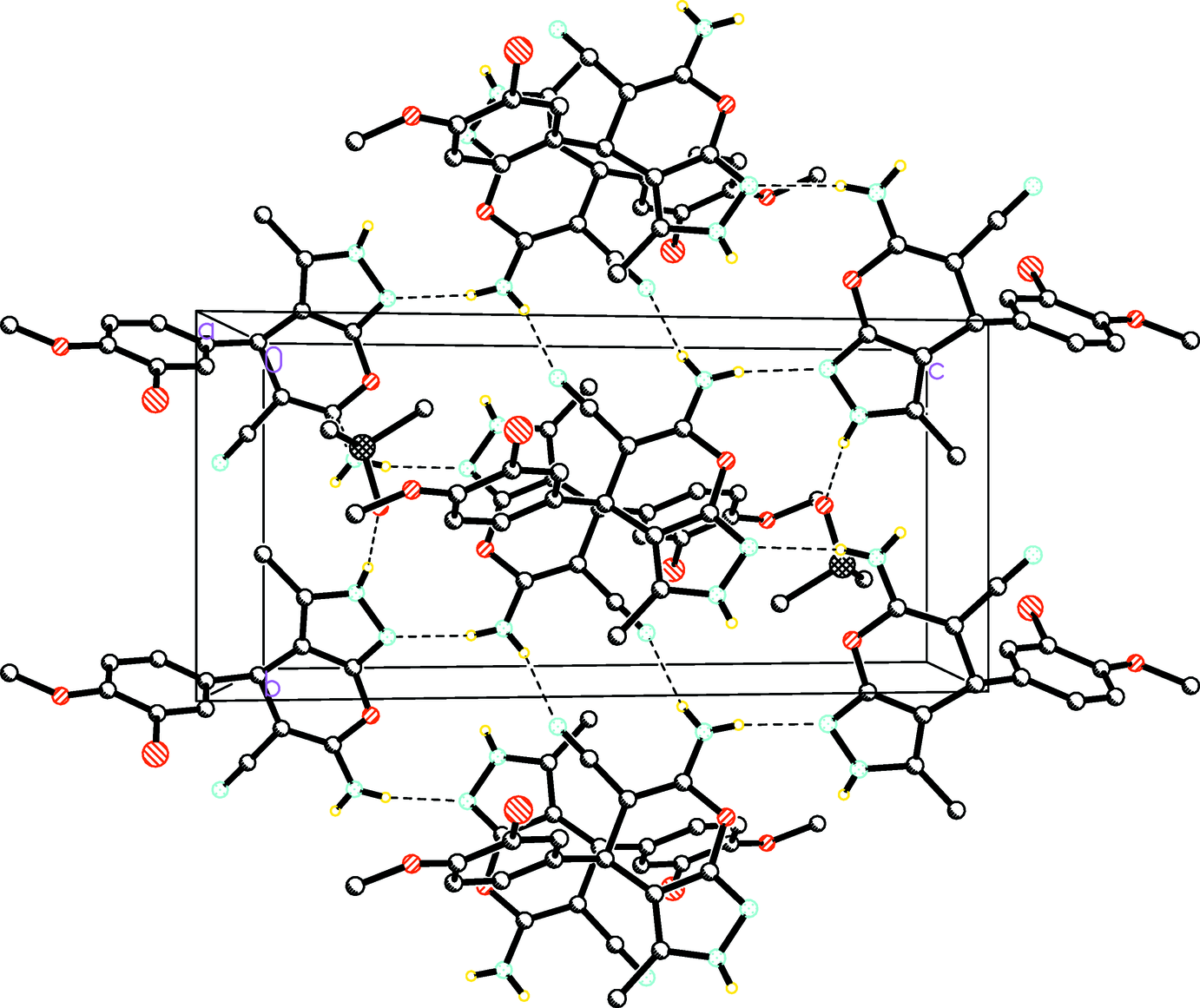

Supplement: Supplementary file 5 [file e-71-0o453-fig2.tif]
